# *COMT* Val158Met Polymorphism and Social Impairment Interactively Affect Attention-Deficit Hyperactivity Symptoms in Healthy Adolescents

**DOI:** 10.3389/fgene.2018.00284

**Published:** 2018-07-31

**Authors:** Sabina K. Millenet, Frauke Nees, Stefan Heintz, Christiane Bach, Josef Frank, Sabine Vollstädt-Klein, Arun Bokde, Uli Bromberg, Christian Büchel, Erin B. Quinlan, Sylvane Desrivières, Juliane Fröhner, Herta Flor, Vincent Frouin, Hugh Garavan, Penny Gowland, Andreas Heinz, Bernd Ittermann, Herve Lemaire, Jean-Luc Martinot, Marie-Laure P. Martinot, Dimitri O. Papadoulos, Tomáš Paus, Luise Poustka, Marcella Rietschel, Michael N. Smolka, Henrik Walter, Rob Whelan, Gunter Schumann, Tobias Banaschewski, Sarah Hohmann

**Affiliations:** ^1^Department of Child and Adolescent Psychiatry and Psychotherapy, Central Institute of Mental Health, Medical Faculty Mannheim, Heidelberg University, Mannheim, Germany; ^2^Department of Cognitive and Clinical Neuroscience, Central Institute of Mental Health, Medical Faculty Mannheim, Heidelberg University, Mannheim, Germany; ^3^Department of Psychosomatic Medicine and Psychotherapy, University of Leipzig, Leipzig, Germany; ^4^Department of Genetic Epidemiology in Psychiatry, Central Institute of Mental Health, Medical Faculty Mannheim, Heidelberg University, Mannheim, Germany; ^5^Department of Addictive Behavior and Addiction Medicine, Central Institute of Mental Health, Medical Faculty Mannheim, Heidelberg University, Mannheim, Germany; ^6^Discipline of Psychiatry, School of Medicine and Trinity College Institute of Neuroscience, Trinity College Dublin, Dublin, Ireland; ^7^University Medical Center Hamburg-Eppendorf, Hamburg, Germany; ^8^Centre for Population Neuroscience and Stratified Medicine and MRC-SGDP Centre, Institute of Psychiatry, Psychology and Neuroscience, King’s College London, London, United Kingdom; ^9^Department of Psychiatry and Neuroimaging Center, Technische Universität Dresden, Dresden, Germany; ^10^Department of Psychology, School of Social Sciences, University of Mannheim, Mannheim, Germany; ^11^NeuroSpin, CEA, Université Paris-Saclay, Gif-sur-Yvette, France; ^12^Department of Psychiatry and Psychology, University of Vermont, Burlington, VT, United States; ^13^Sir Peter Mansfield Imaging Centre, School of Physics and Astronomy, University of Nottingham, Nottingham, United Kingdom; ^14^Department of Psychiatry and Psychotherapy, Charité – Universitätsmedizin Berlin, Berlin, Germany; ^15^Physikalisch-Technische Bundesanstalt, Braunschweig Institute Berlin, Germany; ^16^Institut National de la Santé et de la Recherche Médicale, INSERM Unit 1000 “Neuroimaging & Psychiatry,” Faculté de Médecine, Université Paris-Sud, Le Kremlin-Bicêtre; and Université Paris Descartes, Sorbonne Paris Cité, Paris, France; ^17^Institut National de la Santé et de la Recherche Médicale, INSERM Unit 1000 “Neuroimaging & Psychiatry,” University Paris Sud – Paris Saclay, University Paris Descartes; Service Hospitalier Frédéric Joliot, Orsay; and Maison de Solenn, Paris, France; ^18^Institut National de la Santé et de la Recherche Médicale, INSERM Unit 1000 “Neuroimaging & Psychiatry,” University Paris Sud – Paris Saclay, University Paris Descartes; and AP-HP, Department of Adolescent Psychopathology and Medicine, Maison de Solenn, Cochin Hospital, Paris, France; ^19^Rotman Research Institute, Baycrest Centre for Geriatric Care, Department of Psychology and Psychiatry, University of Toronto, Toronto, ON, Canada; ^20^Department of Child and Adolescent Psychiatry and Psychotherapy, University Medical Centre Göttingen, Göttingen, Germany; ^21^Trinity College Dublin, Dublin, Ireland

**Keywords:** ADHD, *COMT*, social impairment, adolescence, moderation

## Abstract

The dopaminergic system has been shown to have substantial effects on the etiology of attention-deficit hyperactivity disorder (ADHD). However, while some studies found a significant direct effect, others did not. In this context, social behavior might play an important role as a factor that is related both to the dopaminergic system and ADHD. In a large epidemiological sample of adolescents (*N* = 462; 16–17 years), we assessed the level of ADHD symptoms using the Strengths and Difficulties Questionnaire, social behavior using the Social Responsiveness Scale, and the allelic distribution of the dopaminergic catechol-*O*-methyltransferase (*COMT*) Val158Met polymorphism. We found a significant association between *COMT* and social impairment, insofar as Met-allele carriers showed increased levels of social impairment. Moreover, social impairment significantly determined an association between *COMT* and ADHD (explained variance: 19.09%). This effect did not significantly differ between males and females. *COMT* and social impairment might interactively affect ADHD symptomatology, and could thus represent significant gene-phenotypic risk factors for ADHD symptomatology. This might have interesting implications for prevention and intervention strategies with a focus on social behavior in genetically at-risk individuals.

## Introduction

Attention-deficit hyperactivity disorder (American Psychiatric Association [APA], 2013), characterized by inattention, impulsivity, and/or hyperactivity, is one of the most common neurodevelopmental disorders in childhood. In clinical, epidemiological, and behavioral genetic studies impairments in social behavior have frequently been found in ADHD patients (Thapar et al., 2005a; Hoza, 2007; Caspi et al., 2008; McQuade and Hoza, 2008; Andrade and Tannock, 2013; Bunford et al., 2015). Although social impairment has not been considered a core feature of the disorder, it is an important secondary characteristic of ADHD that has implications in real-world functioning (Hoza, 2007; McQuade and Hoza, 2008; Andrade and Tannock, 2014; Bunford et al., 2015) and is identified as a marker for the heterogeneity of the disorder (Caspi et al., 2008). The most pervasive and persistent impairments in children with ADHD are difficulties in peer interactions and experiences of peer rejections (Nixon, 2001; Hoza et al., 2005; Hoza, 2007; Ronk et al., 2011; Janssens et al., 2017). As a consequence, ADHD children might not have enough opportunities to practice social interactions, which can in turn increase socially immature behavior and lead to fewer prosocial skills (Dodge et al., 2003; Hoza, 2007; Tseng et al., 2014).

There is evidence that impairment in social behavior in ADHD is partly determined by common genetic factors (Nadder et al., 2002) for example the catechol *O*-methyltransferase gene (*COMT*) (Thapar et al., 2005b; Caspi et al., 2008). The *COMT* gene is located on chromosome 22q11.2 (Winqvist et al., 1992) and codes for an enzyme involved in one of the major degradative pathways of the catecholaminergic neurotransmitters. One common single-nucleotide polymorphism (SNP) which is due to a guanine to adenine transition at codon 158 and results in a valine-to-methionine substitution leads to a three- to fourfold difference in enzyme activity and as a consequence to a higher dopaminergic state (Lachman et al., 1996). Previous research has demonstrated direct associations between this *COMT* polymorphism and hyperactivity as well as inattention symptoms, traits, and behavior. However, so far, results have been heterogenous. Some studies reported a significant effect of the Val allele (Akutagava-Martins et al., 2016), which was found to be more frequent in children with ADHD compared to healthy control individuals (Qian et al., 2003; Song et al., 2009) or to be related to inferior frontal cortex response to failed inhibitory behavior (White et al., 2014). Others reported a significant effect of the Met allele being preferentially transmitted to ADHD (Qian et al., 2003). The discrepant findings may be partly explained by the use of different analytic approaches in the aforementioned studies.

Interestingly, has not only ADHD been associated with changes in the dopaminergic system, but the regulation of social behavior in general has also been shown to be determined by dopaminergic functioning (Montag et al., 2008; Yacubian and Buchel, 2009; Mier et al., 2010; Skuse and Gallagher, 2011). With respect to COMT, carriers of the Val allele, and thus individuals with enhanced COMT enzyme activity, showed an increase in social cooperative behavior and a stronger response to social interactions and experiences compared to Met/Met-allele carriers (Reuter and Hennig, 2005; Walter et al., 2011). Moreover, dopaminergic augmentation via *COMT* inhibition was found to be associated with increased egalitarian tendencies (Saez et al., 2015).

So far, research has mostly focused on direct associations between ADHD and social problems, almost not considering potential mediating effects. Results of a large epidemiological study in healthy children (Langley et al., 2010) suggest social impairment as an intermediate phenotype explaining the association between *COMT* and antisocial behavior in ADHD. In line with prior findings (Caspi et al., 2008) impaired social understanding mediated the link between COMT and impaired social behavior in children with higher scores of ADHD (Langley et al., 2010). Less efficient processing of the prefrontal cortex (PFC) and a resulting impairment in executive functioning as well as emotional dysregulation were discussed by the authors as the assumed mechanisms underlying this interaction (Caspi et al., 2008). Recently, van Goozen et al. (2016) reported a significant indirect effect of the *COMT* Val allele on aggressive behavior in ADHD patients who were mediated by social/emotional mechanisms, but not by deficits in executive functioning. They specifically identified impaired fear learning and fear empathy as critical risk mechanisms in this context.

Due to the inconsistent findings in the literature, further studies are needed to gain a deeper understanding of the underlying mechanisms and effects of the association between *COMT* and ADHD, and to unravel the possible role of additional factors that might bear any influence of *COMT* on ADHD symptomatology. The investigation of gene–phenotype interactions in this context may add to previous findings and could help to identify vulnerable phenotypes for ADHD symptomatology. Although social behavior has been identified as one critical factor in ADHD, and it is also associated with the dopaminergic system, we have little information on the interaction between dopaminergic genetic predispositions, social impairment, and ADHD symptomatology.

Because of the continuous nature of ADHD symptoms and impairments (Chen et al., 2008; Larsson et al., 2012) we examined the effects of the *COMT* gene Val158Met polymorphism on the degree of ADHD symptoms and on social behavior, as well as their interactions in a large epidemiological sample of adolescents.

## Materials and Methods

### Subjects and Recruitment

The subjects of the present study were part of the European Imaging Genetics (IMAGEN) study (Schumann et al., 2010), a study in a large population-based sample of adolescents. Participants were recruited via school visits, flyers, and residents’ registration offices in Germany, the United Kingdom, Ireland, and France. The present study used data from *N* = 462 adolescents (242 female) at the age of 16–17 years. Data from the sample assessed in France were not analyzed (*N* = 96), because no French validation was available for one of our measures, the Social Responsiveness Scale (SRS) (see below for details) (**Supplementary Figure S1**).

Exclusion criteria for participation in the study were: any mental disorder as defined by the Development and Well-Being Assessment (DAWBA) (Goodman et al., 2000), IQ < 80, alcohol use disorder, serious medical conditions, and previous head trauma with unconsciousness. The study was approved by the local ethics committees and was conducted in accordance with the Declaration of Helsinki. After explaining the study to the adolescents and their legal guardians, written informed consent was obtained.

### Measures

#### Deoxyribonucleic Acid (DNA) Extraction and Genotyping

Deoxyribonucleic acid was extracted from venous blood samples. To ensure high quality and sufficient quantity, DNA extraction was performed by a semi-automated process. All samples were part of a genome-wide genotyping of ∼600,000 autosomal SNPs within the IMAGEN study using the Illumina Quad 610 chips (Illumina, San Diego, CA, United States).

The genotype frequencies of *COMT* Val158Met in the sample were as follows: Val/Val: *n* = 93, Val/Met: *n* = 243, and Met/Met: *n* = 126. The genotype distribution did not differ according to sex (χ^2^ = 3.324, *df* = 2, *p* = 0.190) or site (χ^2^ = 9.800, *df* = 12, *p* = 0.654).

### ADHD Symptoms

To assess ADHD symptom strength, we used the hyperactivity scale from the Strengths and Difficulties Questionnaire (SDQ) (Goodman, 1997), a screening instrument used to detect behavioral and psychosocial problems in children aged 4–17 years. The SDQ is a well-validated instrument, which includes five dimensions: emotional symptoms, conduct problems, hyperactivity/inattention problems, peer problems, and prosocial behavior, which can either be used as continuous variables (scores 0–10) or as categories (for detailed information: www.sdqinfo.org). For the present analyses, we only used the hyperactivity/inattention problem scale, with the total hyperactivity score as dimensional variable [sum of the five hyperactivity items (self-ratings)] (see **Tables 1, 2**).

**Table 1:** Distribution of ADHD symptoms in the current sample.

SDQ-scores for hyperactivity/	Frequency	Original 3-band
inattention problem scale		categorization
0	43	
1	48	
2	76	Normal *N* = 382
3	86	
4	79	
5	50	
6	35	Borderline *N* = 35
7	26	
8	12	Abnormal *N* = 45
9	7	
Total	462	


**Table 2:** Sample characteristics for the whole group and separately for males and females.

	Males (*N* = 220)			Females (*N* = 242)			Total (*N* = 462)		
Age	16.39		(0.43)	16.43		(0.44)	16.41		(0.43)
ADHD symptom strength	3.09		(2.17)	3.67		(2.10)	3.40		(2.15)
Range of ADHD symptom scores		0–9			0–9			
Social impairment	24.78		(15.94)	24.51		(16.45)	24.64		(16.20)
Range of social impairment scores		1–93			0–100			


*Data are reported as means (standard deviation).*

### Social Behavior/Impairment

To measure social behavior/impairment, we used the SRS (Constantino and Gruber, 2005), a widely used, well-validated scale rated by parents/teachers for use in 4–18-year-olds [for details, see Bölte et al. (2008) for the German adaptation and Constantino and Gruber (2005) for the English original version]. The 65 items focus on behavior during the past 6 months and assess engagement in reciprocal social interactions, understanding of emotional and social cues, and motivation to engage with others. Subscales include social awareness, social information processing, capacity for reciprocal social communication, social anxiety, and autistic mannerisms. The total score of social impairment was used as a continuous variable.

To assess the two questionnaires, we used the Psytools software (Delosis Ltd., London, United Kingdom) via its Internet-based platform.

### Statistical Analysis

All analyses were conducted using the Predictive Analytic Software (PASW, SPSS Inc., Chicago, IL, United States) for Windows, version 24.

Effects of COMT on ADHD symptoms and social behavior: To test the direct effect of the *COMT* Val158Met polymorphism on ADHD symptoms and social impairment, we conducted a univariate analysis of covariance (ANCOVA), with ADHD symptoms or social impairment as dependent variables and *COMT* as between-subject factor with three levels (Val/Val, Val/Met, and Met/Met), adjusting for sex and conduct problems as potential confounding variables. Data were also corrected for multiple comparisons.

Interaction of *COMT*, social behavior, and ADHD symptoms: In a subsequent moderator analysis (Preacher and Hayes, 2008), we tested the impact of social impairment as a moderator of any effect of *COMT* on ADHD symptoms using the *COMT* Val158Met genotype as independent variable (predictor) and ADHD symptoms as dependent variable (outcome) (Baron and Kenny, 1986). Sex and conduct problems were again used as covariates. Using this analysis, associations between the predictor and the outcome, the predictor and the moderator, and the outcome and the moderator are investigated, and it is assumed that the associations between the predictor and the outcome significantly depend on a third variable, the moderator [evaluated via the Sobel (1982) test]. This analysis is based on *a priori* hypotheses and was thus also performed in the case of non-significant effects of *COMT* on ADHD symptoms (e.g., according to Shrout and Bolger, 2002).

For all analyses, *p*-values < 0.05 are reported.

## Results

### Impact of *COMT* Genotype on ADHD Symptoms and Social Impairment

**FIGURE 1:**
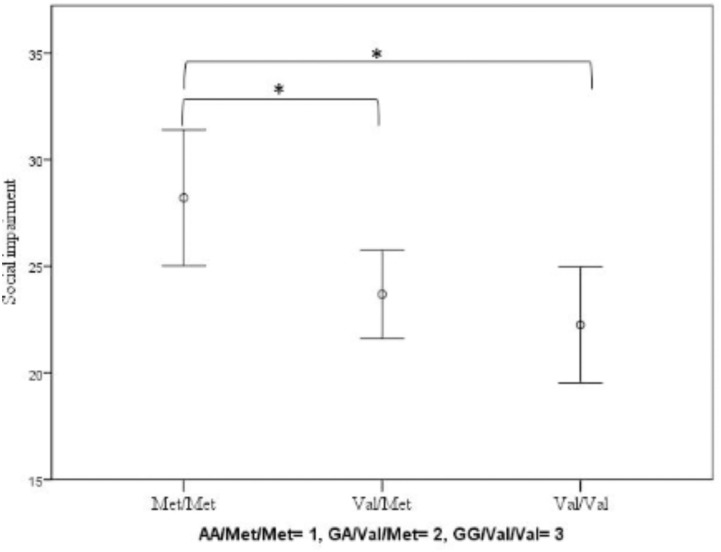
Impact of *COMT* Val158Met genotype on social impairment measured by the SRS. Means and error bars ±2 standard errors (SE) are shown. Significant ^∗^*p* < 0.05.

We found no significant effect of *COMT* on ADHD (*F*_(2.460)_ = 1.537; *p* = 0.216; partial eta-squared = 0.007; covariates: sex: *F*_(1.460)_ = 16.248; *p* < 0.001; partial eta-squared = 0.034; conduct problems: *F*_(1.460)_ = 94.844; *p* < 0.001; partial eta-squared = 0.172). However, *COMT* did have a significant impact on the level of social impairment (*F*_(2.460)_ = 4.376; *p* = 0.013; partial eta-squared = 0.019; covariates: sex: *F*_(1.460)_ = 0.026; *p* = 0.873; partial eta-squared = 0.000; conduct problems: *F*_(1.460)_ = 22.941; *p* < 0.001; partial eta-squared = 0.048): Homozygote Met-allele carriers showed increased levels of social impairment compared to Val-allele carriers (**Figure 1**).

### Interaction of *COMT*, ADHD Symptoms, and Social Impairment

**FIGURE 2:**
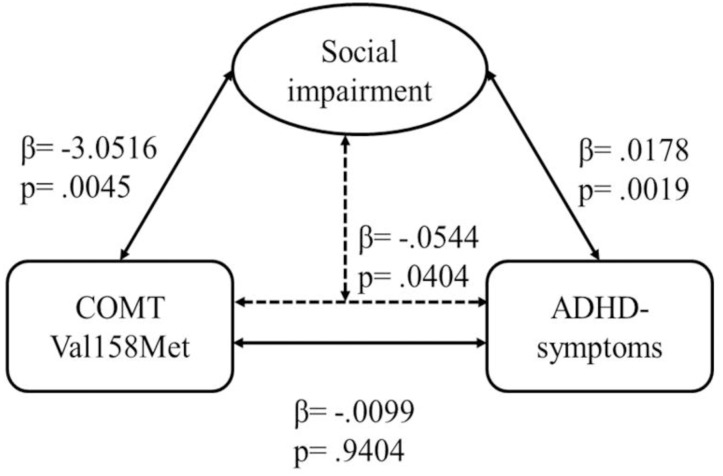
Moderation analysis for effects of *COMT* Val158Met genotype on ADHD symptom strength via social impairment. The association between *COMT* and ADHD symptom strength was significant when the measure of social impairment was included in the model; Sobel test: *p* = 0.0404.

The regression model with *COMT* as predictor, ADHD symptoms as dependent variable, and social impairment as moderator was significant [*F* = 29.834; *p* < 0.001; explained variance of ADHD symptoms: *R*^2^ = 19.09%; female: 13.55% (*p* < 0.001); male: 22.32% (*p* < 0.001)]. There was a direct effect of *COMT* on social impairment (β = -3.0516; *t* = -2.8557; *p* = 0.0045), and of social impairment on ADHD symptom strength (β = 0.0178; *t* = 3.1175; *p* = 0.0019), but no effect of *COMT* on ADHD symptoms (β = -0.0099; *t* = -0.0748; *p* = 0.9404) (**Figure 2**). However, we found an indirect effect of COMT on ADHD moderated by social impairment (β = -0.0544; *Z* = -2.0492; *p* = 0.0404), and significant effects of the two covariates (sex: β = 0.7060; *t* = 3.9237; *p* = 0.0001; conduct problems: β = 0.5757; *t* = 8.9919; *p* < 0.0001).

## Discussion

The dopaminergic system has substantial effects on ADHD etiology. However, so far, studies have yielded inconsistent results, with some finding a significant association and others failing to do so. These discrepant findings may be attributable to a significant effect of further factors such as social behavior, which might modulate the direct association between *COMT* and ADHD, and thus represent a significant moderator driving the influence of the dopaminergic system on ADHD symptomatology. In the present study, we found no significant association between the dopaminergic *COMT* polymorphism and ADHD symptoms, although this might depend on social impairment: The level of social impairment served as a moderator of the association between *COMT* and the levels of ADHD symptoms. Moreover, *COMT* was further directly significantly associated with social impairment.

While previous studies found positive associations of both the Val and the Met allele with ADHD symptomatology (Qian et al., 2003; Song et al., 2009); several meta-analyses reported no significant association between ADHD and Val158Met (Cheuk and Wong, 2006; Gizer et al., 2009; Lee and Song, 2015; Bonvicini et al., 2016), which is in line with our findings. Moreover, studies have yielded mixed results with respect to sex: Some reported effects of *COMT* Val158Met on ADHD and related symptoms or traits only in boys, while others observed the opposite results. In the present study, we did not observe any significant difference between male and female participants. This indicates that social impairment is an important general facet of ADHD that is strongly triggered by the catecholaminergic system, but not so much by sex-related biological or social constraints.

In our study, we observed a significant effect of the Met/Met genotype on social impairment, insofar as Met/Met carriers showed increased levels of impairment. Thus, higher synaptic dopamine levels following neurotransmitter release may increase social impairment and related problematic behavior. Dopaminergic systems are related to neural networks that support attentional control, salience detection, and self-referential cognition, and are associated with high levels of intrinsic motivation and reward (e.g., Gangi et al., 2016; Di Domenico and Ryan, 2017). Moreover, the Met compared to the Val/Val allele genotype has been shown to be related to increased trait anxiety (Montag et al., 2008) and higher loss-aversion behavior (Schmack et al., 2008). Such behavioral changes in, for example, responsiveness to punishment have also been found in ADHD (Tsang et al., 2015; Furukawa et al., 2017; Ruf et al., 2017). *COMT* and its associated functional consequences may thus serve as critical neurobiological determinants for ADHD-related risk and problem behavior. By contrast, compared to the Met genotype, the Val genotype was associated with positive emotionality and extraversion (Reuter and Hennig, 2005), and may thus serve a protective function in reducing ADHD symptomatology and related problems.

Beyond ADHD, the role of *COMT* is investigated in conjunction with many other mental disorders (Taylor, 2018). Moreover, social impairment even if it is immanent for ADHD (Ros and Graziano, 2018), it is not specific for ADHD, but also present in many other mental disorders (American Psychiatric Association [APA], 2013; WHO, 2016). Therefore, one further possible explanation for the association between *COMT*, social impairment, and ADHD might be that the association is not specific for ADHD but associated with a possible general impact resulting from mental disorders. This would be in line with the RDoC approach which aims in understanding the nature of mental health/illness not in diagnostic categories but in terms of dysfunction in general psychological systems.

Previous studies have reported that not only children with ADHD but also individuals with ADHD symptoms at subthreshold diagnostic levels have problems with peers (Hoza, 2007; McQuade and Hoza, 2008) and more often experience peer rejection, friendship problems, and peer neglect (Hodgens et al., 2000; Bagwell et al., 2001; Diamantopoulou et al., 2007; Willcutt et al., 2012). Moreover, symptoms of ADHD such as intrusiveness and salience are suggested to have an important impact on peer functioning (Pope et al., 1989; Diamantopoulou et al., 2007; Andrade and Tannock, 2013, 2014), and predicted social problems at a 2-year follow-up period (Humphreys et al., 2016). The dopaminergic system may play a critical role in this context, as it was also shown to be related to social learning (e.g., Diaconescu et al., 2017) and social motivation (Gunaydin and Deisseroth, 2014). Our present results add to these findings by demonstrating that social impairment serves as a significant moderator of ADHD symptoms in individuals who are genetically at risk through higher dopaminergic functioning. In genetically high-risk individuals, adequate behavioral assistance and guidance with respect to social relationships may thus be an important factor in order to improve ADHD symptomatology. This could be realized, for instance, through interventions to target the interpretation of affective cues to assist processes of social decision-making (Humphreys et al., 2016).

As one limitation of the present study we have to mention that our sample sizes (*N* = 462) is rather small for a genetic association study; thus, we were not able to further subdivide the sample to perform a replication or sex-related analyses. It could be speculated that a direct effect between *COMT* and ADHD – against conclusion from three meta-analyses (Cheuk and Wong, 2006; Gizer et al., 2009; Lee and Song, 2015) – could be detected in a larger sample with sufficient power. Therefore, results must be independently replicated in at least on other clinical as well as non-clinical sample and until then should be viewed as tentative. One could also criticize that we focused in our analysis on only one genetic variant and did not include other SNPs reported to be of relevance in ADHD. We based our hypothesis on former reports concerning the influence of *COMT* on antisocial behavior in ADHD patients (Thapar et al., 2005a; Caspi et al., 2008) and possible mediating effects (Langley et al., 2010; van Goozen et al., 2016) and thus chose to only investigate *COMT* effects in this context. However, it would also be of interest to include other dopaminergic genes or genes that have been identified by GWAS in ADHD samples in further investigations using larger clinical or non-clinical samples. Moreover, finding has to be treated with caution due to a possible overestimation of the genetic effect (Lohmueller et al., 2003) based on the used SNP analysis. In sum, a significant interrelation between *COMT*, ADHD symptoms, and social impairment in an epidemiological sample of adolescents was observed. This indicates significant gene–environment risk factors for ADHD symptomatology also at subthreshold levels, and could inform strategies to prevent or manage social problems in daily life in genetically at-risk individuals.

## Author Contributions

AB, CBü, HG, PG, AH, J-LM, TP, MR, MS, HW, RW, GS, TB, and HF study design. SM, FN, SHo, SHe, CBa, SV-K, UB, EQ, JUF, and HL data assessment and recruitment. SM, FN, JOF, SD, VF, BI, M-LM, DP, LP, and TB data management and analyses. SM, FN, SHo, and TB paper writing.

## Conflict of Interest Statement

TB has served as an advisor or consultant to Bristol-Myers Squibb, Desitin Arzneimittel, Eli Lilly, Medice, Novartis, Pfizer, Shire, UCB, and Vifor Pharma; he has received conference attendance support, conference support, or speaker’s fees from Eli Lilly, Janssen McNeil, Medice, Novartis, Shire, and UCB; and he is involved in clinical trials conducted by Eli Lilly, Novartis, and Shire; the present work is unrelated to these relationships. SM received conference attendance support from Shire. SHo, SHe, SV-K, AB, UB, CB, EQ, SD, HF, VF, HG, PG, AH, BI, J-LM, M-LM, DP, TP, LP, JF, MS, HW, RW, GS, and FN report no financial relationships with commercial interests.

## Supplementary Material

FIGURE S1 Differences between French data and data from the other IMAGEN centers were also found in the subscales social cognition, social communication, social motivation, and social awareness. Excluded French data: *N* = 96.
