# Supplementary figures and images for: COMT Val158Met Polymorphism and Social Impairment Interactively Affect Attention-Deficit Hyperactivity Symptoms in Healthy Adolescents
Source: Front Genet. 2018 Jul 31;9:284. doi: 10.3389/fgene.2018.00284 (PMC6079264; doi:10.3389/fgene.2018.00284)

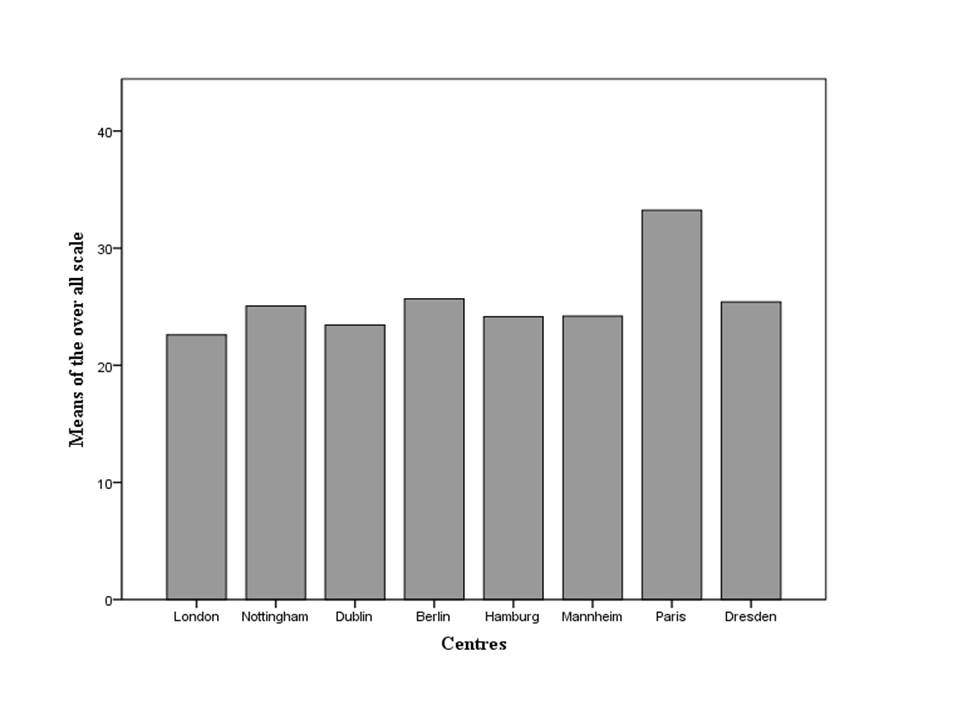

Supplement: FIGURE S1 — Differences between French data and data from the other IMAGEN centers were also found in the subscales social cognition, social communication, social motivation, and social awareness. Excluded French data: N = 96. [file Image_1.JPEG]
